# LcNAC13 Physically Interacts with LcR1MYB1 to Coregulate Anthocyanin Biosynthesis-Related Genes during Litchi Fruit Ripening

**DOI:** 10.3390/biom9040135

**Published:** 2019-04-04

**Authors:** Guoxiang Jiang, Zhiwei Li, Yunbo Song, Hong Zhu, Sen Lin, Riming Huang, Yueming Jiang, Xuewu Duan

**Affiliations:** 1 Guangdong Provincial Key Laboratory of Applied Botany, South China Botanical Garden, Chinese Academy of Sciences, Guangzhou 510650, China; gxjiang@scbg.ac.cn (G.J.); lizhiwei@scbg.ac.cn (Z.L.); ybsong@scbg.ac.cn (Y.S.); zhuhong@scbg.ac.cn (H.Z.); ymjiang@scbg.ac.cn (Y.J.); 2 Key Laboratory of Post-Harvest Handling of Fruits, Ministry of Agriculture, Guangzhou 510650, China; 3 University of Chinese Academy of Sciences, Chinese Academy of Sciences, Beijing 100049, China; 4 School of Biomedical Engineering, Wenzhou Medical University, Wenzhou 325027, China; lin_sen@wmu.edu.cn; 5 Guangdong Provincial Key Laboratory of Food Quality and Safety, College of Food Science, South China Agricultural University, Guangzhou 510642, China; huangriming@scau.edu.cn

**Keywords:** litchi, anthocyanin biosynthesis, NAC, R1MYB, transcriptional regulation

## Abstract

Anthocyanin accumulation is crucial for the development of quality for most fruit. The mechanism underlying the regulation of anthocyanin biosynthesis by transcription factors in litchi fruit remains largely unknown. In this study, we isolated one NAC (NAM, ATAF1/2 and CUC2) TF gene, *LcNAC13*. Expression of *LcNAC13* was upregulated as ripening proceeded, followed by the accumulation of anthocyanins. Electrophoretic mobility shift assay (EMSA) and transient expression assay showed that LcNAC13 could negatively regulate the expression of anthocyanin biosynthesis-related genes, including *LcCHS1/2*, *LcCHI*, *LcF3H*, *LcF3’H*, *LcDFR*, and *LcMYB1*. Furthermore, LcR1MYB1, as one R1-MYB type MYB, was identified to physically interact with LcNAC13 and reverse the effect of LcNAC13. Taken together, these results suggested that LcNAC13 and LcR1MYB1 may act together to antagonistically regulate anthocyanin biosynthesis during litchi fruit ripening, which helps to provide new insights into the regulatory networks of anthocyanin biosynthesis.

## 1. Introduction

Anthocyanins are a class of flavonoids with >250 members widely distributed in plants [1]. Anthocyanins constitute the main pigments in flowers and fruits, resulting in the typical red, blue, purple, or black color characteristics, and contributing to the marketable quality of some vegetables and fruits [2,3]. Anthocyanins play important biological roles in the growth, development, and responses to the environmental stimuli in plants, not only endowing flowers and fruits with various colors to attract pollinators and seed distributors [4], but also acting as antioxidants that scavenge reactive oxygen species (ROS) and function in pathogen defense responses [5,6,7,8]. More importantly, anthocyanins are the health-promoting components in the human diet due to their antioxidant properties [9], which reduce cardiovascular diseases, cancer, neurodegenerative disorders, cataracts, and inflammation [10,11].

The anthocyanin biosynthesis pathway has been studied extensively and genes involved in most of the biosynthetic steps have been characterized in various plants [12,13]. Anthocyanin biosynthesis is catalyzed by numerous enzymes, including phenylalanine-ammonia lyase (PAL), 4-coumaryl:CoA ligase (4CL), chalcone synthase (CHS), chalcone isomerase (CHI), flavanone 3-hydroxylase (F3H), flavonoid 3’-hydroxylase (F3’H), dihydroflavonol 4-reductase (DFR), anthocyanidin synthase (ANS), flavonol 3-glucosyltransferase (3GT), and rhamnosyl transferase (RT), which are encoded by structural genes [3,14]. Transcription of these structural genes are controlled in some species by regulatory genes, such as MYB transcription factors (TFs), basic helix-loop-helix (bHLH) TFs, and WD40-repeat (WDR) TFs. These TFs form a ternary complex of MYB-bHLH-WD40 TFs (MBW complex), which has been recognized as the key factor to regulate anthocyanin accumulation in plants [15,16]. Recently, other transcription factors (TFs) have been found to be involved in the transcriptional regulation of anthocyanin biosynthesis-related genes. For example, MdEIL1 positively regulates anthocyanin biosynthesis in apple fruit via direct binding to the promoter of *MdMYB1* [17], whereas MdHB1, as a negative regulator of anthocyanin biosynthesis in white-fleshed ‘Granny Smith’ via recruiting MBW TFs to the cytoplasm, indirectly suppresses the transcription of *MdDFR* and *MdUFGT* [18]. In Arabidopsis, ANAC078 positively regulates anthocyanins biosynthesis during high-light conditions [19], whereas ANAC032 negatively regulates anthocyanin biosynthesis during stress conditions [20]. In addition, PpNAC1 positively regulates peach fruit anthocyanin biosynthesis during ripening process [21]. Therefore, the anthocyanin biosynthesis in plants is regulated by multiple transcription factors. Considering the complex of transcriptional regulation of anthocyanin biosynthesis, other key players in the complicated regulatory network remain to be identified and elucidated.

Litchi (*Litchi chinensis* Sonn.) is a subtropical fruit with high commercial value on the international market due to its delicious taste and attractive red peel [9,22]. Anthocyanins constitute the basis of the red color of litchi pericarp. The major red pigments in litchi pericarp include cyaniding-3-rutinoside and cyaniding-3-glucoside [23]. Anthocyanins are synthesized during fruit development and degraded after fruit are harvested. Anthocyanin synthesis and degradation are crucial for the development and maintenance of sensorial quality of litchi fruit. Previous studies indicated that *LcMYB1* positively regulates litchi fruit anthocyanin biosynthesis-related genes’ expression (*LcF3H*, *LcF3’H*, *LcUFGT*, *LcDFR*, *LcANS*, *LcGST*) during fruit coloration, which are well correlated with the elevated anthocyanin content [24,25]. In addition, LcbHLH1/3 and LcSPL1 interact with LcMYB1 to coregulate anthocyanin biosynthesis and fruit coloration [25,26]. However, the mechanism underlying the regulation of anthocyanin biosynthesis in litchi fruit is still largely unknown.

In the present study, we reported two TFs, namely LcNAC13 and LcR1MYB1, which are involved in regulating anthocyanin biosynthesis-related genes during litchi fruit ripening. LcNAC13 directly bound to the promoters of anthocyanin biosynthesis-related genes, including *LcCHS1/2*, *LcCHI*, *LcF3H*, *LcF3’H*, *LcDFR* and *LcMYB1*, and repressed their transcription, while LcR1MYB1 physically interacted with LcNAC13 and opposed the negative effect, suggesting that LcNAC13 and LcR1MYB1 might act together to regulate anthocyanin biosynthesis-related genes during litchi fruit ripening. Our findings revealed a novel transcriptional regulation mechanism of anthocyanin biosynthesis in plants.

## 2. Materials and Methods 

### 2.1. Plant Materials and Treatments

Ten-year-old litchi (*Litchi chinensis* Sonn. cv. Huaizhi) trees were used for the study from a local orchard in Guangzhou, China. ‘Huaizhi’ litchi is widely cultivated in South China, rich in anthocyanins, which is a good material for investigating the regulation mechanism of anthocyanins biosynthesis in fruit. Litchi fruit were randomly sampled from different parts of the canopy in the morning. Fruit samples were taken initially 50 days after flowering (DAF) and at four successive 10 d intervals during development and ripening (five sampling times in all, i.e., 50 DAF, 60 DAF, 70 DAF, 80 DAF, and 90 DAF). Fresh fruit was used for the analysis of fruit width, average fruit weight, total chlorophylls content, total flavonoids, anthocyanins, and cyanidin-3-rutinoside. The pericarp was collected, frozen in liquid nitrogen, and stored at −80 °C for RNA extraction. All trees used in the experiment were maintained according to commercial litchi production practices.

### 2.2. Flavonoid and Chlorophyll Measurement

Chlorophyll level was analyzed according to the protocol of Arnon [27]. Total anthocyanins content in pericarp was measured by a pH-differential method [28]. Flavonoid level was assayed as previously described [29] and cyanidin-3-rutinoside content was extracted and determined by high performance liquid chromatography (HPLC) according to the protocol of Zhang et al. [30].

### 2.3. RNA Extraction and Quantitative Real-Time PCR Analysis

Total RNA was extracted using the hot borate method [31] and the cDNA was synthesized by using PrimeScript™ RT reagent Kit with gDNA Eraser (Takara, Otsu, Japan). qRT-PCR was performed with SYBR^®^ Premix Ex Taq TM II (Takara, Otsu, Japan) in an ABI7500 Real-Time PCR System (Thermo Fisher Scientific, Waltham, MA, USA). Relative levels of the gene transcripts were quantified by normalizing to the *LcACT1* gene using 2^−ΔΔCT^method [32]. The gene-specific oligonucleotide primers used for qPCR analysis are described in Supplementary Table S1. Three independent biological replicates were used in the analysis.

### 2.4. Promoter Isolation

Genomic DNA was extracted from litchi pericarp using the DNeasy Plant Mini Kit (Qiagen, Valencia, CA, USA). The promoters of *LcCHS1/2*, *LcCHI*, *LcF3H*, *LcF3’H*, *LcDFR*, *LcANS*, *LcLAR* and *LcMYB1* associated with flavonoid biosynthesis [33], were isolated (Supplementary Figure S1) using a Genome Walker Kit (Clontech, Mountain View, CA, USA) with nest PCR according to the manufacturer’s instructions. The amplification products were cloned into pGEM-T Easy vector (Promega, Madison, WI, USA) and sequenced. Conserved cis-element motifs of promoter were predicted using PLACE (http://www.dna.affrc.go.jp/PLACE/signalscan.html) and Plant-CARE (http://bioinformatics. psb.ugent.be/webtools/plantcare/html/) databases.

### 2.5. Electrophoretic Mobility Shift Assay (EMSA)

The oligonucleotide probes corresponding to the promoters of the above seven anthocyanin biosynthesis-related genes were synthesized and labeled with DNA 3′ End Biotinylation Kit (Thermo Fisher Scientific, Waltham, MA, USA). The His-LcNAC13 fusion protein and the biotin-labeled fragments were used for EMSA. The unlabeled DNA fragment was used as a competitor. The EMSA was performed using the EMSA kit (Thermo Fisher Scientific, Waltham, MA, USA) according to the manufacturer’s instructions.

### 2.6. Yeast Two-Hybrid (Y2H) Assay

The coding sequences of *LcNAC13* and *LcR1MYB1* were subcloned into pGBKT7 or pGADT7 vector to fuse with the DNA-binding domain (DBD) and activation domain (AD), respectively, to create the bait and prey. Then, the different pairs of bait and prey constructs were cotransformed into yeast strain AH109 by the lithium acetate method, and yeast cells were grown on DDO medium (minimal media double dropouts, SD medium with -Leu/-Trp) according to the manufacturer’s protocol (Clontech, Mountain View, CA, USA) for 3 days. Transformed colonies carrying the indicated vectors were plated onto QDO medium (minimal media quadruple dropouts, SD medium with -Leu/-Trp/-Ade/-His and 15 mM 3-AT), to test the possible interaction between LcNAC1 and LcR1MYB1 according to their growth status.

### 2.7. Expression and Purification of Recombinant LcNAC13 and LcR1MYB1

The complete coding region of *LcNAC13* was cloned into pET-28a vector (Novagen, Madison, WI, USA) to generate His-LcNAC13, while the *LcR1MYB1* cDNA fragment was inserted into pGEX-4T-3 (Amersham Biosciences, Staffanstorpm, Sweden) to fuse in frame with GST. His and GST recombinant fusion proteins were expressed in BL21 (DE3) cells with induction by 1.0 mM isopropyl-b-D-thiogalactoside for 12 h at 16 °C. The recombinant proteins were then purified with Ni^2+^-nitrilotriacetate (Ni-NTA) agarose (Qiagen, Valencia, CA, USA) and Glutathione Sepharose 4B (GE Healthcare, Pittsburgh, PA, USA) according to the manufacturer’s manual, respectively.

### 2.8. GST Pull-Down Assay

The GST-pull down assay was performed as described previously [34]. Purified His-LcNAC13 was incubated with GST or GST-LcR1MYB1 bound to glutathione Sepharose 4B beads (GE Healthcare, Pittsburgh, PA, USA). The eluted proteins were subjected to SDS-PAGE and Western blotting. Gel blots were analyzed using anti-His antibody and anti-GST (Abcam, Cambridge, MA, USA) antibodies. The chemiluminescent signal was visualized with enhanced chemiluminescence reagent (Pierce, Rockford, IL, USA).

### 2.9. Bimolecular Fluorescence Complementation (BiFC) Assay

Full-length coding sequences of LcNAC13 and LcR1MYB1 without stop codons were cloned into pUC-pSPYNE or pUCpSPYCE vectors. The resulting constructs were transiently expressed in *Arabidopsis* mesophyll protoplasts by modified PEG transfection [35]. After incubation at 22 °C for 24–48 h, yellow fluorescent protein (YFP) was observed using a Zeiss Axioskop 2plus confocal laser scanning microscope (Leica, Solms, Germany).

### 2.10. Co-IP Assay

Full-length coding sequences of LcNA13 and LcR1MYB1 were cloned into pCAMBIA-1302 and myc-PBA vectors to create the LcNA13-GFP and LcR1MYB1-myc constructs, respectively. The resulting constructs were transformed into Agrobacterium tumefaciens strain GV3101 and infiltrated into tobacco (*Nicotiana benthamiana*) leaves. After culture for 3 d, the leaves were ground in liquid nitrogen and resuspended in 10 mL extraction buffer (50 mM Tris-HCl pH 7.5, 150 mM NaCl, 5 mM EDTA, 2 mM dithiothreitol supplemented with 10% glycerol, 1% polyvinylpolypyrolidone, 1 mM phenylmethylsulfonyl fluoride, plant protease inhibitor cocktail). After centrifugation at 12,000× *g* and 4 °C for 20 min, the supernatant was incubated with 30 μL GFP-Trap^®^_A beads (ChromoTek) at 4 °C for 4 h, and the beads were washed four times with wash buffer (50 mM Tris-HCl, pH 7.5, 250 mM NaCl, 5 mM EDTA, 10% glycerol, 1 mM phenylmethylsulfonyl fluoride). The protein complexes were eluted from the beads by boiling with 2× SDS sample buffer, and analyzed by SDS-PAGE and immunoblotted with anti-GFP (Abcam, Cambridge, MA, USA) and anti-Myc (Abcam, Cambridge, MA, USA) antibodies.

### 2.11. Subcellular Localization Analysis

The coding regions of *LcR1MYB1* and LcNAC13 without a stop codon were subcloned into the pCAMBIA-1302 vector, respectively. The resulting constructs were introduced into *Agrobacterium tumefaciens* strain GV3101 and infiltrated into tobacco (*Nicotiana benthamiana*) leaves. The infiltrated leaves were then incubated at 22 °C for 24–48 h. YFP fluorescence was observed using a Zeiss Axioskop 2plus florescence microscope (Leica, Solms, Germany). All transient expression assays were repeated at least three times.

### 2.12. Dual-Luciferase Reporter Assay (DLR)

In accordance with a previous protocol [34], the open reading frames of *LcNAC13* and *LcR1MYB1* were fused to pGreenII 62-SK as effector plasmids, respectively. The promoters of the above eight anthocyanin biosynthesis-related genes were inserted into pGreenII0800-LUC as reporter plasmids, respectively. The above constructs were transiently expressed in tobacco leaves by *Agrobacterium*-mediated infiltration (strain GV3101). Luciferase assays were performed using the Promega dual-luciferase reporter assay system and a Luminoskan Ascent Microplate Luminometer (Thermo Fisher Scientific, Waltham, MA, USA) 3 d after co-transformation. The transcriptional activity of the TFs on the promoters were indicated by the ratio of LUC/REN. At least six biological replicates were assayed for each combination.

### 2.13. Data Handling

Experiments were performed in completely randomized design. Data were expressed as mean ± standard error. Differences among different treatments were analyzed and compared at the 5% level using SPSS version 7.5 (IBM SPSS, Armonk, NY, USA).

## 3. Results

### 3.1. Physiological Characteristics of Litchi Fruit during Ripening

**Figure 1:**
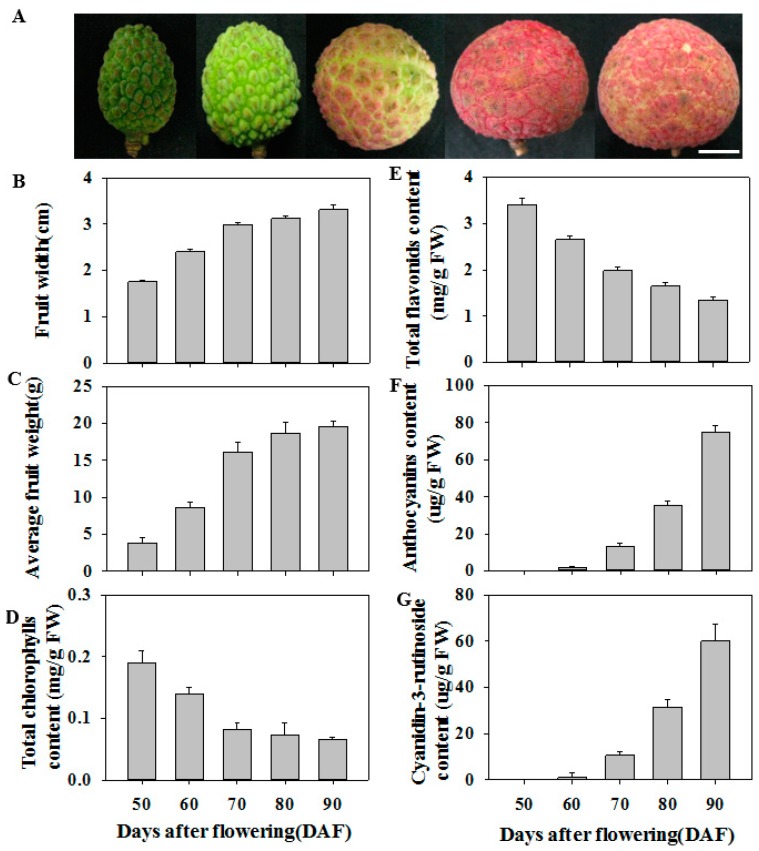
Visual appearance (**A**), fruit width (**B**), average fruit weight (**C**), total chlorophylls content (**D**), total flavonoids content (**E**), total anthocyanin content (**F**), cyanidin-3-rutinoside content (**G**) of litchi fruit during ripening. Left to right: fruit at 50 days after flowering (DAF), 60 DAF, 70 DAF, 80 DAF, and 90 DAF. Bar = 1 cm. Each value represents the mean + standard error (n = 20 in B and C; n = 3 in D–G).

Figure 1A shows the appearance of litchi fruit at different development stages from 50 d to 90 d after flowering (DAF). The fruit growth and development were obviously divided into two stages according to average fruit width (Figure 1B) and fruit weight (Figure 1C): a rapid growth phase (50–70 DAF) and a slow growth phase (70–90 DAF). Chlorophyll content in litchi pericarp decreased rapidly at 50–70 DAF, but was almost constant at 70–90 DAF (Figure 1D). Total flavonoids content in litchi pericarp gradually decreased as the fruit developed (Figure 1E). Anthocyanins in litchi pericarp could not be detected in litchi pericarp at 50 DAF, but subsequently rapidly increased at 60–90 DAF. Moreover, the content of cyanidin-3-rutinoside, the major anthocyanin in ‘huaizhi’ litchi fruit, shows similar trend to total anthocyanins content, which accounted for more than 85% of the total anthocyanins content (Figure 1F,G). Total anthocyanins content increased in parallel with the increase in pericarp red color during ripening.

### 3.2. Expression Profiles of LcNAC Genes during Litchi Fruit Ripening 

**Figure 2:**
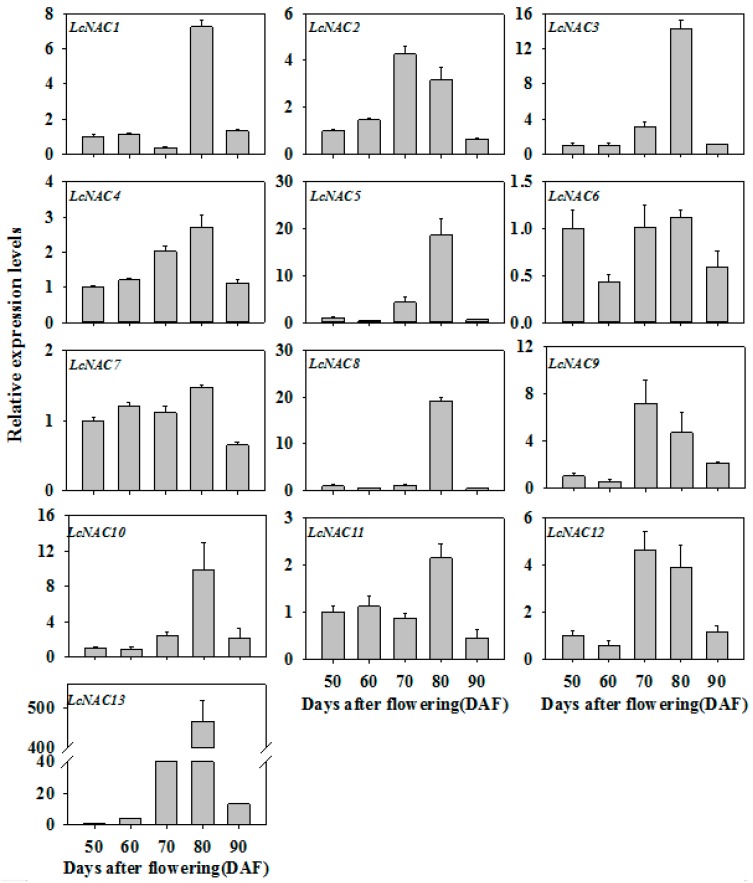
Expression of 13 *LcNAC* genes in litchi fruit pericarp during ripening. The expression levels are expressed as a ratio relative to that at 50 DAF, which was set as 1. *LcACT1* was used as an internal control. Each value represents the mean + standard error of three replicates.

To understand the possible role of *LcNAC* genes in litchi fruit development and ripening, and anthocyanin biosynthesis, the transcript levels of *LcNACs* in litchi pericarp during development and ripening were investigated by quantitative real-time PCR (qRT-PCR). The transcript levels of *LcNAC1/3/4/5/8/10/11/13* genes were significantly upregulated when fruit turned red, i.e., from 70 DAF to 80 DAF. Of these genes, *LcNAC13* showed the most significant upregulation (Figure 2), which was in consistent with the accumulation of anthocyanins (Figure 1). In contrast, the transcript levels of *LcNAC2/6/7/9/12* genes decreased or changed only slightly as anthocyanins accumulated. Therefore, we selected LcNAC13 as a candidate gene to further study its function in anthocyanin biosynthesis in litchi fruit.

### 3.3. LcNAC13 Binds to the Promoters of Anthocyanin Biosynthesis-Related Genes 

**Figure 3:**
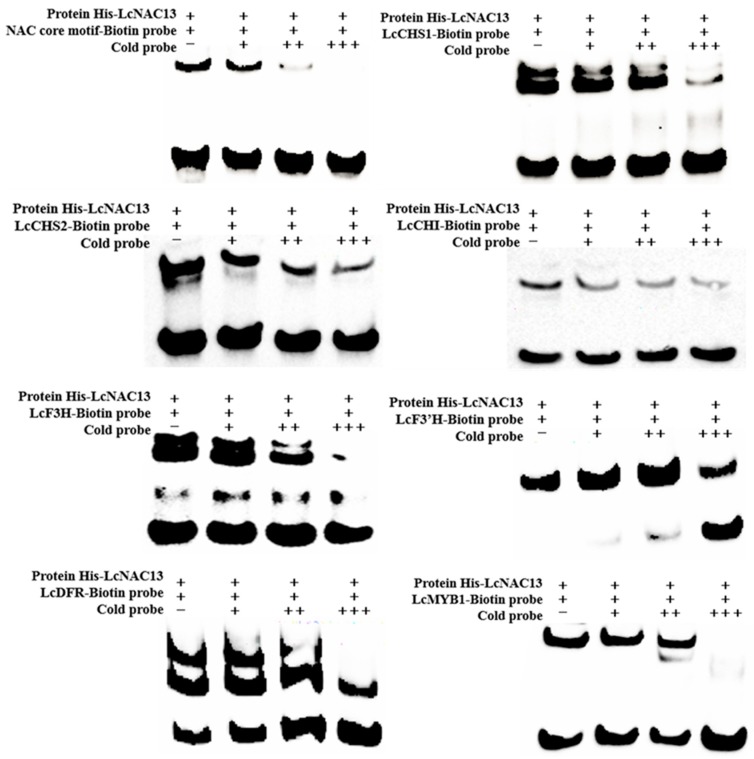
Interaction between LcNAC13 and the anthocyanin biosynthesis-related genes in vitro. Purified His-LcNAC13 recombinant proteins were mixed with biotin-labeled probes, and the DNA–protein complexes were separated on 6% native polyacrylamide gels. +, ++, and +++ indicate increasing amounts (100 nM, 10 μM, and 50 μM) of unlabeled probes for competition.

NAC proteins can recognize the NAC core motif present in the promoters of target genes [36]. We examined whether LcNAC13 can bind specifically to the NAC core motif using electrophoresis mobility shift assays (EMSA). As shown in Figure 3, a protein–DNA complex with reduced mobility was detected when LcNAC13 protein was incubated with the NAC core motif probe, and the binding of LcNAC13 to the NAC core motif was reduced when unlabeled probe was added, suggesting that LcNAC13 specifically bound to the NAC core motif in vitro. Furthermore, LcNAC13 proteins could strongly bind to the NAC core motif-containing promoters of *LcCHS1/2*, *LcCHI*, *LcF3H*, *LcF3’H*, *LcDFR*, and *LcMYB1*, which have been reported to be involved in anthocyanin biosynthesis during the stage from color-breaker to red ripe [24,29,33].

### 3.4. LcNAC13 Physically Interacts with LcR1MYB1

**Figure 4:**
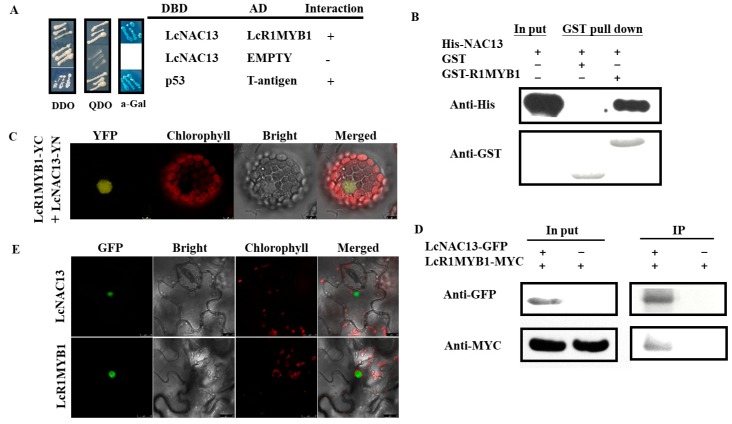
Interaction between LcNAC13 and LcR1MYB1 in vitro and in vivo. (**A**) Interaction between LcNAC13 and LcR1MYB1 in the Y2H assay. (**B**) Interaction between LcNAC13 and LcR1MYB1 in vitro in the GST pull-down assay. (**C**) Interaction between LcNAC13 and LcR1MYB1 by bimolecular fluorescence complementation in Arabidopsis mesophyll protoplasts. (**D**) Interaction between LcR1MYB1 and LcNAC13 in the coimmunoprecipitation assay. (**E**) Subcellular localization of LcNAC13 and LcR1MYB1 in tobacco (*Nicotiana benthamiana*) leaves. Green signal indicates GFP fluorescence; yellower signal indicates YFP fluorescence; red signal indicates chlorophyll autofluorescence. The merged images represent digital combination of the chlorophyll autofluorescence and YFP fluorescent images or GFP fluorescent images. Scale bar = 10 μm.

To investigate the role of LcNAC13 in anthocyanin biosynthesis in litchi fruit, we performed an Y2H screening using LcNAC13 as bait to identify its interacting proteins from a litchi fruit cDNA expression library. We identified 24 positive colonies. Among the positive colonies, one cDNA corresponding to *LcR1MYB1* was most frequently identified, which was selected for further study. Both Y2H analysis (Figure 4A) and GST pull-down assay (Figure 4B) verified the interaction between LcNAC13 and LcR1MYB1. Moreover, the BiFC analysis confirmed that LcNAC13 interacted with LcR1MYB1 in vivo (Figure 4C; Supplementary Figure S2). We also performed a coimmunoprecipitation (Co-IP) assay using *N. benthamiana* leaves transiently expressing GFP/LcR1MYB1-myc or LcNAC13-GFP/LcR1MYB1-myc. The LcR1MYB1-myc fusion proteins were detected after immunoprecipitation of LcNAC13-GFP (Figure 4D), further indicating that LcNAC13 could interact with LcR1MYB1 in vivo. In addition, a subcellular localization analysis indicated that LcNAC13 and LcR1MYB1 were targeted to the nuclear compartment (Figure 4E).

### 3.5. Expression Profiles of LcR1MYB1 during Litchi Fruit Ripening

**Figure 5:**
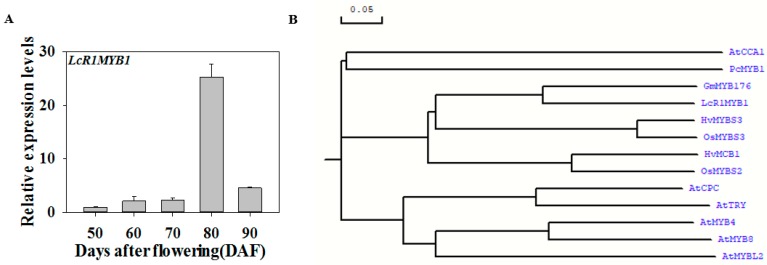
(**A**) Expression of *LcR1MYB1* in litchi fruit pericarp during ripening. The expression levels of *LcR1MYB1* is expressed as a ratio relative to that at 50 DAF. Each value represents the mean + standard error of three replicates. (**B**) Phylogenetic analysis of LcR1MYB1and R1-MYBs from different species. Phylogenetic tree was constructed was constructed by the neighbor-joining method of DNAMAN6.0. The scale bar represents 0.05 substitutions per site. The amino acid sequences were obtained from NCBI with the accession numbers as below: AtMYBL2 (AEE35154), AtTRY (AED96321), AtCPC (AEC10691), AtMYB4 (NP_195574.1), AtMYB8 (XP_002891188.1), GmMYB176 (ABH02865), OsMYBS2 (AAN63153), OsMYBS3(AAN63154), PcMYB1(AAB61699.1), HvMYBS3(CAJ53899.1), HvMCB1(CAI84066.1).

The transcript level of *LcR1MYB1* was apparently upregulated at the stages of fruit enlargement and color-breaker, which was consistent with fruit development and the change in color of the pericarp. The upregulation of *LcNAC13* was earlier than that of *LcR1MYB1* (Figure 2 and Figure 5A). Furthermore, bioinformatics analysis indicated that LcR1MYB1 shared a close relationship with GmMYB176 (Figure 5B; Supplementary Figure S3), which affects isoflavonoid synthesis in soybean by regulating *CHS8* gene expression [37,38]. These results implied that LcNAC13 and LcR1MYB1 might be involved in the regulation of litchi fruit anthocyanin biosynthesis.

### 3.6. The Repressive Effect of LcNAC13 on Anthocyanin Biosynthesis-Related Genes is Reversed by LcR1MYB1 in Transient Expression Assays

**Figure 6:**
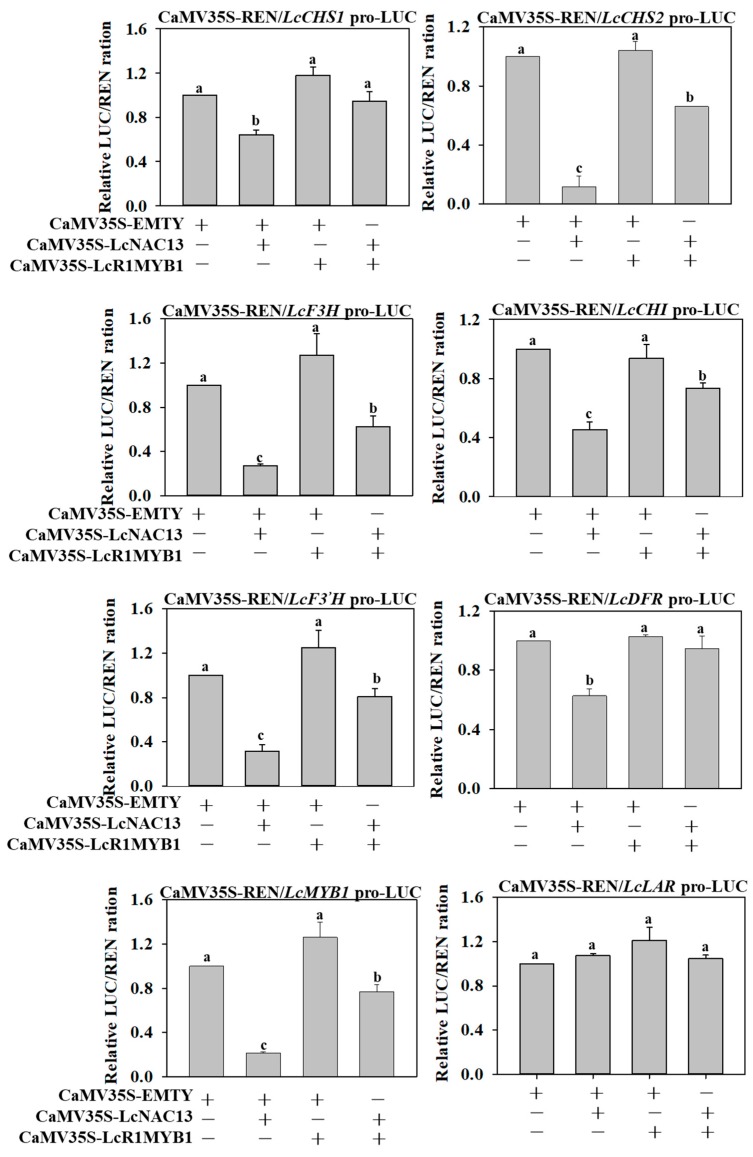
Transient expression assays demonstrate the function of LcNAC13 and LcR1MYB1 in transcriptional regulation of anthocyanin biosynthesis-related genes in vivo. Repression or activation of LcNAC13 or LcR1MYB1 to the promoters of anthocyanin biosynthesis-related genes were shown by the ratio of LUC to REN. The ratio of LUC to REN of the empty vector plus promoter vector was used as a calibrator (set as 1). Different letters above the bars represent a difference (*p* < 0.05). Each value represents the mean + standard error of six biological replicates.

As above mentioned, transcript levels of *LcNAC13* and *LcR1MYB1* were significantly upregulated when fruit turned red, which was in consistent with the accumulation of anthocyanins, and LcNAC13 physically interacted with LcR1MYB1. We hypothesized that the interaction between LcNAC13 and LcR1MYB1 cooperatively regulated the expression of anthocyanin biosynthesis-related genes in litchi fruit. To verify the hypothesis, we performed transient expression assays in tobacco leaves using the dual-luciferase reporter system. As shown in Figure 6, coexpression of LcNAC13 with *LcCHS1/2*, *LcCHI*, *LcF3H*, *LcF3’H*, *LcDFR*, or *LcMYB1* significantly decreased the LUC/REN ratio, while coexpression of LcNAC13 with *LcLAR* (as a control) showed no effect on the LUC/REN ratio, suggesting that LcNAC13 trans-repressed anthocyanin biosynthesis-related genes. However, the trans-repression was reversed when LcR1MYB1 was coexpressed (Figure 6). These data indicated that LcR1MYB1 likely acted as a repressor of LcNAC13 in regulating expression of anthocyanin biosynthesis-related genes.

### 3.7. Expression Patterns of LcNAC13-Targeted Genes are Correlated with the Change in Pericarp Color during Fruit Ripening

**Figure 7:**
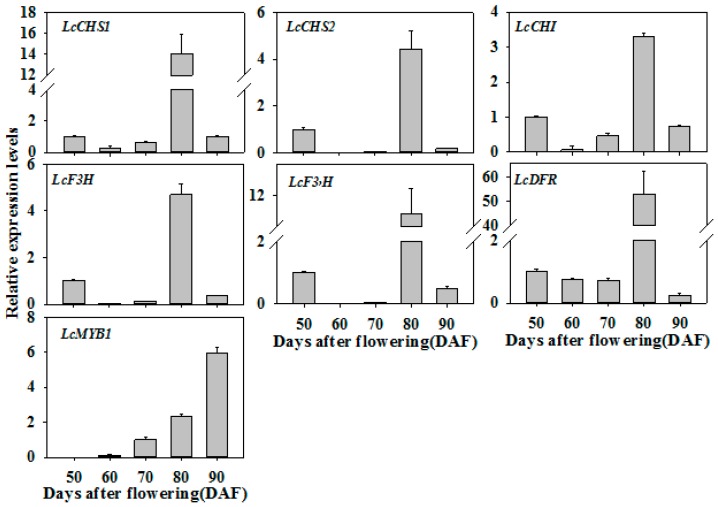
Expression of anthocyanin biosynthesis-related genes in litchi fruit pericarp during ripening. The expression levels of *LcCHS1/2, LcCHI, LcF3H, LcF3’H*, and *LcDFR* are expressed as a ratio relative to that at 50 DAF, while the expression level of *LcMYB1* is expressed as a ratio relative to that at 70 DAF. Each value represents the mean + standard error of three replicates.

Expression patterns of anthocyanin biosynthesis-related genes (*LcCHS1/2*, *LcCHI*, *LcF3H*, *LcF3’H*, *LcDFR* and *LcMYB1*) in the pericarp during fruit development and ripening are shown in Figure 7. Expression of *LcCHS1/2*, *LcCHI*, *LcF3H*, *LcF3’H*, and *LcDFR* genes tended to downregulation or showed no change as the fruit developed toward pigmenting. At 80 DAF, expression of *LcCHS1/2*, *LcCHI*, *LcF3H*, *LcF3’H* and *LcDFR* were significantly upregulated, accompanied by rapid accumulation of anthocyanins and fruit coloring. Expression of *LcMYB1* was significantly upregulated throughout development and ripening stages, especially during fruit ripening. 

## 4. Discussion

Anthocyanins are important health-promoting pigments and make a major contribution to the quality of fruits. Anthocyanins are structurally composed of anthocyanidin aglycon and sugar moieties [12]. The most frequently occurring anthocyanidins in fruits are cyanidin, delphinidin, pelargonidin, peonidin petunidin, and malvidin. In general, genetic characteristics determine anthocyanin type present in fruits [3]. In ripe ‘huaizhi’ litchi fruit, the major anthocyanin was cyanidin-3-rutinoside, which accounted for more than 85% of the total anthocyanin content [39]. Developmental and environmental factors play keys roles in regulating anthocyanin biosynthesis in fruits [3]. In the present study, no or lower level of anthocyanins were found during the rapid growth phase (50–70 DAF), while anthocyanins were rapidly synthesized during the slow growth phase (70–90 DAF), indicating that anthocyanin synthesis in litchi fruit was a process regulated by developmental factor (Figure 1). 

Numerous studies demonstrate that transcription factors mediate developmental and environmental regulation of anthocyanin biosynthesis in fruits [3]. Transcriptional factors directly manipulate the expression of structural genes in the anthocyanin biosynthetic pathway. NACs are one of the largest families of plant-specific transcription factors. The involvement of NACs in plant growth, development, and stress response have been well studied [40,41,42,43]. An increasing number of NAC transcription factors have been identified as senescence regulators in higher plants [44]. In addition, NAC transcription factors are involved in the regulation of climacteric fruit ripening and nonclimacteric fruit senescence. Litchi is a typical nonclimacteric fruit. In the present study, expression of 13 *LcNAC* genes were significantly upregulated during fruit development. Of the 13 *LcNAC* genes, transcript of *LcNAC13* gene at 80 DAF increased more than 450 times compared with that at 50 DAF (Figure 2), which was consistent with the change in total anthocyanin content. The results implied that LcNAC13 possibly was implicated in the regulation of anthocyanin biosynthesis in litchi fruit. 

The involvement of NACs in the regulation of anthocyanin biosynthesis have been reported. In Arabidopsis, ANAC078 positively regulates anthocyanin biosynthesis during high-light conditions [19], whereas ANAC032 negatively regulates anthocyanin biosynthesis during stress conditions [20]. PpNAC1 positively regulates peach fruit anthocyanin biosynthesis during the ripening process [21]. However, in these studies, NACs do not directly regulate the structural genes in the pathway of anthocyanin biosynthesis, and only act as activators or repressors to regulate the function of anthocyanins biosynthesis-related transcriptional factors. Previous studies indicated that the expression of *LcCHS1/2*, *LcCHI*, *LcF3H*, *LcF3^’^H*, *LcUFGT*, *LcDFR*, *LcGST*, and their regulatory gene *LcMYB1* are all upregulated in litchi fruit during fruit coloration, which were well correlated with the elevated anthocyanin content [24,25,33]. In the present study, LcNAC13 could directly bind to NACs motifs of the anthocyanin biosynthesis-related genes and regulate their transcription, including *LcCHS1/2*, *LcCHI*, *LcF3H*, *LcF3^’^H*, *LcDFR*, and *LcMYB1* (Figure 3). Moreover, LcNAC13 regulated anthocyanin accumulation during litchi fruit ripening by transcriptional repression of *LcCHS1/2*, *LcCHI*, *LcF3H*, *LcF3^’^H*, *LcDFR*, and *LcMYB1* (Figure 6). Our results indicated that LcNAC13 was involved in regulation of anthocyanin biosynthesis by directly mediating the structural and regulatory genes expression. 

The MYB family is another one of the richest groups of transcription factors in plants, with key roles in regulating development, metabolism, and stress response [45,46,47,48,49]. MYB proteins are characterized by a highly conserved MYB domain consisting of one to three imperfect repeats. MYB can be divided into three major categories based on number of repeats: R1R2R3-MYB, R2R3-MYB, and R1-MYB. The R2R3-MYB represents the most abundant class of MYB. The regulation of anthocyanin biosynthesis is mainly related to this type of MYB. R2R3-MYB in combination with bHLH and WD40, forming the MYB-bHLH–WD40 protein complex, directly regulates the expression of the structure genes in the pathway of anthocyanin biosynthesis [15,16]. Numerous R2R3-MYB transcription factors have been reported to be implicated in the regulation of anthocyanin biosynthesis in peach, strawberry, apple, and pear [17,21,45,47,50,51]. In these reports, most of the R2R3-MYBs act as positive regulators that activate the expression of the structural pathway genes. In litchi fruit, LcMYB1, the homologs of Arabidopsis MYB75/PAP1 and MYB90/PAP2, is shown to positively regulate anthocyanin biosynthesis and fruit coloration by directly targeting the downstream anthocyanin-associated genes [24]. In addition, LcbHLH1/3 and LcSPL1 interact with LcMYB1 to coregulate anthocyanin biosynthesis and fruit coloration [25,26]. Although several R3-MYB transcription factors have been reported to act as negative regulators of anthocyanin biosynthesis [52,53,54], there is a relative paucity of information on the involvement of R1-MYB in regulating the structural genes in the pathway of anthocyanin biosynthesis. In the present study, we isolated one R1-MYB gene, named *LcR1MYB1*. Coexpression of *LcR1MYB1* with *LcCHS1/2*, *LcCHI*, *LcF3H*, *LcF3’H*, *LcDFR*, or *LcMYB1* resulted in no significant changes in the expression of these target genes (Figure 6), suggesting that LcR1MYB1 could not individually regulate the expression of the structural genes in the pathway of anthocyanin biosynthesis. 

Interactions between TFs or TFs and proteins to form enhanceosome or repressosome complexes are important regulatory mechanisms of gene expression, including anthocyanin biosynthesis-related genes [55,56,57,58]. For example, PyMYB114 physically interacts with PyERF3 to coregulate anthocyanin biosynthesis and fruit coloration [57]. Similarly, FaERF#9 and FaMYB98, through their physical interactions, activate transcription of *FaQR* and upregulate HDMF biosynthesis in strawberry [58]. In the present study, LcR1MYB1 could physically interact with LcNAC13 and cooperatively regulate the expression of anthocyanin biosynthesis-related genes (*LcCHS1/2*, *LcCHI*, *LcF3H*, *LcF3^’^H*, *LcDFR*, and *LcMYB1*) during litchi fruit ripening (Figure 6). Our results implied the antagonistic interaction between NAC and R1MYB in a transcription complex. Aside from the interaction between LcNAC13 and LcR1MYB1, whether this transcription complex influenced the anthocyanin biosynthesis by interfering with MWB complex requires investigation. 

## 5. Conclusions

In summary, two transcriptional factors, LcNAC13 and LcR1MYB1, were identified to be involved in the regulation of anthocyanin biosynthesis-related genes during litchi fruit ripening. LcNAC13 directly bound to the promoters of anthocyanin biosynthesis-related genes (*LcCHS1/2*, *LcCHI*, *LcF3H*, *LcF3’H*, *LcDFR*, and *LcMYB1*) and repressed their transcription, while LcR1MYB1 physically interacted with LcNAC13 and reversed the negative effect of LcNAC13. This is the first report regarding the involvement of NAC in the regulation of anthocyanin biosynthesis via direct regulation on structural and regulatory genes. Moreover, based on previous studies and our results, in addition to the mechanism of MYB-centered regulation of anthocyanin synthesis in litchi fruit, other transcription factors such as NAC may also be directly involved in the transcription regulation of anthocyanin synthesis. More transcription factors and regulatory networks in relation to the regulation of anthocyanin biosynthesis require further investigation.

## Supplementary Materials

The following are available online at https://www.mdpi.com/2218-273X/9/4/135/s1, Table S1: Primers used for gene cloning and quantitative real-time PCR analysis, Figure S1: Nucleotide sequence of the promoters used in this work, Figure S2: Bimolecular fluorescence complementation analysis, Figure S3: Sequence alignment of LcR1MYB1 with other plant R1-MYB proteins.
