# Supplementary material for: LcNAC13 Physically Interacts with LcR1MYB1 to Coregulate Anthocyanin Biosynthesis-Related Genes during Litchi Fruit Ripening
Source: Biomolecules. 2019 Apr 4;9(4):135. doi: 10.3390/biom9040135 (PMC6523642; doi:10.3390/biom9040135)
Supplement: Supplementary file 1 [file biomolecules-09-00135-s001.pdf]

Supplementary material

Article

LcNAC13 physically interacts with LcR1MYB1 to co-regulate anthocyanin biosynthesis-related genes during litchi fruit ripening

Guoxiang Jiang[1,2], Zhiwei Li[1,3],Yunbo Song[1], Hong Zhu[1], Sen Lin[4], Riming Huang[5], Xuewu Duan[1,2*], Yueming Jiang[1,2]

[1] Guangdong Provincial Key Laboratory of Applied Botany, South China Botanical Garden, Chinese Academy of Sciences, Guangzhou 510650, China; gxjiang@scbg.ac.cn (G.J.); lizhiwei@scbg.ac.cn (Z.L.); ybsong@scbg.ac.cn (Y.S.);zhuhong@scbg.ac.cn; ymjiang@scbg.ac.cn (Y.J.)

[2] Key Laboratory of Post-Harvest Handling of Fruits, Ministry of Agriculture, Guangzhou 510650, China

[3] University of Chinese Academy of Sciences, Chinese Academy of Sciences, Beijing 100049, China

[4] School of Biomedical Engineering, Wenzhou Medical University, Wenzhou 325027, China. lin_sen@wmu.edu.cn

[5] Guangdong Provincial Key Laboratory of Food Quality and Safety, College of Food Science, South China Agricultural University, Guangzhou 510642, China; huangriming@scau.edu.cn

* Correspondence: xwduan@scbg.ac.cn

Table S1. Primers used for gene cloning and quantitative real-time PCR analysis

**Assay**	**Gene**	**Forward (5'-3')**	**Reverse (5'-3')**
RT-PCR	*LcNAC13*	ATGGTTCACACGAAGAACCCTGAAT	TCCTTGGAACTGGAGATGAGGGCTC
	LcR1MYB1	ATGAATAATCTGTCTCAGTATGAAC	TCAAGCAACACTGATAATGCTACCG
qRT-PCR	*LcR1MYB1*	ACCTGCCTTCCCTGTAGTATTG	TCGGACGGATGAGATTTGC
	LcCHS1	CCATCATTGTTGGGTCAGA	TGAGGAGGTGGAATGTGAG
	LcCHS2	GACATTGTGGTGGTGGAGGT	TATTTAGCGAGACGGAGGAC
	LcCHI	CCTCTATCCTTTTCACACAATC	CGTCTTCAGGCTTCTTATCAT A
	LcF3H	CTGCAACTCTCACAGCTCTT	GAAGATACCCCAGTCCTCAC
	LcF3'H	GTGAGATACTGGAGGAGCATAA	TACAGTGCTTGATGTGGTGTC
	LcDFR	ATAAAGCCAACTATCAATGGGAT	AGCCCATATCACTCCAGCAAGT
	LcMYB1	GTTGGTCCCTTCAATCTTATC	GAAGACGAGGACTCCAACAC
	LcNAC1	CTTCTATGCCGCAGGTTT	TCACTGTCTAAACCCGAAC
	LcNAC2	GATGATTGGGTATTGTGC	TGACTTTGGACCTCCTTATT
	LcNAC3	GAGGTTGCTCCTGTTTGTC	GACTGGCTCAGGAAGGATA

LcNAC4	AATGGAGCCTATTGAGC	GCGACTGGAGGATGTTAA
LcNAC5	CAAGGGATGGTGAGATTAGG	TAGAAAGGTGGAGGAGCAAG
LcNAC6	AGAAGCCCATCATAGGCACTCG	CCGTCACTAAATGGAGGGTTGG
LCNAC7	GCAGTAAGCGGTCAAGGATA	GTCACAGGCAGTACCACAAG
LcNAC8	TTCCATAGAGGCAAAGCACCAA	TGTCATCATCATCATCCCAATC
LcNAC9	CCCAACTGGAAACAAACCTCA	TCATCCCAGTTTCCATTCAC
LcNAC10	AGAGCACCGACTCGTCTACC	CGGATACTTCCTGTCCCTTG
LcNAC11	CTTTCCACGCATCTCCTTCT	GCTGTTGCCAGTTCTTGTTG
LcNAC12	GCTACCAAACGGGTCTGAAT	TGAGAATTGCTCCACCTTGC
LcNAC13	CGTGGAGAACAAACTCAAGCG	TTGGAACTGGAGATGAGGG

<*LcCHS1*-promoter

ATAGGAACTTATTTCATGGGTATATTTTTTTTCTTCAATTTAAAAAATTTGTAAATGTT
TCTAATAACTATATCATTGTAATAATTCTGGATTAGAAATGGGAAGGATTTTATAGTC
ATAGAAAAGTCAGTTTTTTCTTAGAAGCAAGGCAAACTATTCAACTACCGAATTCAAG
TGTTTTATTTAATTTGATATCTTTTTACTATGGCTTCCTTACTTTCACAATATTGGGGAG
AGGTTTTTCCATACCTTTTAGTAAATATGAAATATAAGACTTGAATCTTTCATCTCAAA
TATAAAAGAGAAATTACACATCACCTAGGCAAAATTTAGGTTAGTTTTACAATATTTA
GTTGTAGGTGTAATGGTTCTTTGACATTAAAAAATATTGGAATATAAGCATAAAGTTA
TTTTGAATCAAGAAAAACTTTATCATCCCTTTGACCAATAGCAAAAATAACAATGTAA
TTTGGAATAAATAAAATTTTTCTGCAAAGGTTTTTAACTAATTTCATAATCATATGATT
ATTTCTTAATTTTAATCGATCTTAGGGGGCTTTGAAATTCTCTCTTGAATTTGAAACTA
GACATGGATATCAATAATAAAAATCAACCAATAAAGTAGGGATTGGAGATTTCCATG
GGATATCTATAATATAAACAACCACTAGCAAGGGATGATTATTATCATTGTGGCTAAG
GTAGCAGGAGACTTTGTCTAAGTTCTTAACTAATTTTAAAGTTGTATGGTTGTTTGTCA
ATTTTAGTTAAGACTAAAGGGCTGTTTTGAAATTTGCCCTTGAATATAAAATGATTCA
TTAATGCGATATAAATTAACCATTAAATTAACTAAAGACTAAAACCCCATGCGCAGG
AGGACTTTTTACCTGTTGTTTCGACAGGACAGCGAGGACTCTTAGTTTCAAAGTAATG
TAATTATATTTTCAGTTGTACTCAAATCTGAAAGAGAATTTTTTCCCTTAAATCTAAAA
TGCAACAAGATTTTAATACAAATCATGCATCAAACTACAGATTTTTCTGAAGATTTCA
TCGGATCATATTAGTAAGTAATACAGAATTATTTGGTTTAGAATCCAAGTAAATTATT
ACATTTACTGAATTATAATTTTATCAACATAAATAGAAAGTTGATACTTGTTATAGTA
AATTTCCAAGTAAATTATTGATCTCATTTAAGAAGTTACCGTCTAATTTTATTTTATTT
TCTTTTCATATAAAAGTTATCTCAAAATTGAAAATCCTATAAGATCTATGCTAATATA
AGACATAACCTTTATGCCTTACCTTAAAAAGAGGCTACTCATTGGGGTCACTATTAAT
TACCTATCATTCCTTTGTTGTGTAATCCTCCTAACATTACACATTATGGGTCACACCTT
TTCATAAAATTACTTATTAATGTGTGATTTTTAGAAACTTAATACAAATTTTGACTATA
TACACATATCATGTTTTGAATTATTGAAGAAAAATTTCAGAACTTACTTGACAATAAA
CACAGTTATTAATTTACGTATACCTTAAGAAAGCTCGTGATAATCCACAAATGAAAAT
ATAAAATCCTAGTTTGTAAACTAATTCATGAATTGTGGCCATTCAATTGTCTTGACTA
ATTGCATCAGTACCTTGATGAATGAACCTAATGTGGTAAGTGAGAAGAGCTTGCTAG
CTACAACTCCATATCATGTGGAGGATAGACAGGCAAGTAA**GCGAACGTTTTATAGA**
TAGATACACCAAACACCTTTGTCACGTGCCAGCCAGCGGCTGCCCAACCGAGTTGT
TGAGTCATTTTGGATCTTGTACAACTATCGGATTGGTCGATCACGTGATCTTCAGCTA
CCCACATCTCCCACTCTTATATATGTGCAACTAAAACATTTTTTTTTCATACTACTTC
CTCTCTCACACATACAAAATCTCTAAGACCTAGCAGCTGGTCCCTCGATTGTGATCAA
AACTGCACCACAGGATCTTCACAGCCAGTTTTGATCAGTTTTTCTCTTGAAGCTAGGG
AAAAATG

<*LcCHS2*-promoter

TGTTTATTATACTGAATTTAAATGAGTCAGTCTCAACTCATATTTCAAACTTCTATCTT
AATAAATGTTATGGTACCTGGTGAATATATATTTTTGTAAGTGAAATTAATTGTTATC

AATGTATATACATTCACCAATTGCTACATCATTAATTGGATGCATGTATGATATGAAA
TGTGAGATGTCCAATATTATTTAATTTAAATATATTCTTTTTTTTTGAAACATTAATTT
AAATATATTCAAACCAAGTTTATTAAAAATAAATTCAATCTAACCAATATAAACATAA
TTAATATATTGAAAGTCGTTTAATTAACTACTTTTGTCTATATAATCCATAATACAAAA
ATAAAAGTATATTAAGATATGAAATTATTGTATATAGTAACATGTAAGTTACACGTAT
ATTGTTAAATTCTAAATTTACCTTTAATAATTATGTTGAACTTGTAAATCCTTAAGCAA
ATTTGACACGGTTTGTTTGATAAATGAGTTAAACGGGTTGACGTAATTAGGGGAATCA
AAAATTGAAAATTATCTATTTCTTGTTTGTATTGAATTGTACGTTATACTGTGGCTCAA
TGATCCAAACTATGACATCTATGATCTATCTAATACAATTATCTTGAATTTATATATAA
TTTAAACTCATAACTGAAAAGCTCATTAAATATAATACAAACGTCATTCAATTTAATT
ATAAATGTCTTGTTGCATTGTTTGTAATCTTGATTATTTGGTATATTCTTTGTTATAAA
GGGCATATTGTATATTATTACTGTCATGTGCTATTTTATAAGTTGAAGTTGGCATTCAA
CGATACTTTTTAATTATTTCAATAAATAGACGGTTATTTCTATGAAACAGTTCTAAATT
TCTACTTATACAAAATAGGAAGAATACTTTGCGTTAGTTGACTTCGTAGCGTATTTCTT
CGATATATGTGTGTATTTCCATTACATATTTTCTGGTAGTCAAATGAACAAACATTTG
GACATAATTTGGTCCTGTCAAGATCAAAACTTGTAAGCAAAAATCAAAAACTAAAAA
ACTAAAATAAGTCGTAAGTATTTTCAATTAATTAATTAATTAGATTCAAGTAATTTTC
AGAAGTATTATTAGAATAACCTAATTTGTCTTGTTTTATTAATATGACAAAATTTGAT
GATCCAGTTAATTTCTAAAGCAATTACAAGACTCTTTTAATTGTCTTTTTATTATTATT
ATTATTAATTTCCTATTCCAAAATAGTAACCTTGGATTATATATTTTTCTTTACCGTAA
TGTCTGAAATATAAAAGAAAAAGAATTTGTGATTCAATATAATTAGTTTTCTTGAAAG
TTGTTTAATCTTGAATTTGGGTTTTTAGG**TAGATGGCGTGATCATGTGACGTGATAA**
ACCATTTTTAGACCTATCAATCTACAATACTTTGAAATATATATATTCCTCAAATAGG
TGTTAAAGCGATACCTGATAGAATTCTAAAATTACTTCAATTTAAATCCGGACATTAA
TTATCTCAACAGAGATACATGTACAATTTTCTTGATCTTTCTTTGAAGATAAGTTAATT
TCTTCAATTGAAGTAACTTAATCGTACGAATTAATTGAACCAAGTAAACATGCATGAC
ATAAATCGACATGTATTGACGATACATGAAACATGGAAGATGAAACGAGATGTGGCA
TCACAACAACCACTGCTTAATGAGCCTAACACCGAGCCCACGTGGTGACGAGCTGCC
CATTGATCCAACCTACCACTCCCCTCCTCCTTTAAAACCCTACTCAACTAGATTTCCTA
ACATCTCACTCACCTCCTTTCAAGTACGAGCCAAGCTTCAGGCAGGTACTTTCATTTTT
TATCACTAAGCACAAAAATG

<*LcCHI*-promoter

CAGAAAATTCCGCATTGTAGTTGCTCAGCGATAAGCTTTAGCGCCCTACGT
GCGATGCAGTAAATTCCGTAACGTAGTGGTTGAGATAACTTAGTAAGCTT
CACGTTGGCGTTAGTTTTAATCGCCCGTGATGGTTAGAATGTTTAAAGGAT
ATACATGGGGGTTTTGAAAGAAATCATAAAACAAATGATAAATTTTCATG
TTAGTGTATGTGATATTATATCTTCGTTGCACTAACTTATTTGTTTATTTTG
TAGCTATTTATATTTAATTGTGTGCCACTGCGGACGACAGGTCGTCATCAT
TTTGTTGTATATATATAGGTGGGCGATCTATGATATATGATGATGTAAAAC
TTTATGGATGAGATGAATAAGTAATTGAGAAACAAATATTCATATTTTGA
AATTGTATATTTGTAAATATTCATGCATTTGATATATATGATGCATCTAGA

TATATTCTACATGAGTTCGTGCTTATGAAAAGAGAATTGAGTGTTGATGTT
GCATGTTTGAGAATAAAGAAGTTGGGAGGAATGAAATGGGTTGGGAATA
AAGAAGTTGGGAGGAATTGAGGTTTTAGTCGAAGTGTAAATTTCTTGACA
TGTAAATTTTGGTTTATCAAAAATGCAGCCGTTATACTGCCGAAAATTTTA
TAAAAATATTTGAAATAGAATCGAGAAAAATTTTATCACTCCCAACTAGT
TTAAAAGAAAACTGAATTTTTGAGAACAAACGAGTAAACCGGTTGAAGAA
AGTTAGGGTTTGCTTCTCGGATTTCTTCGATCCGACAAGTCAGTTTCGATA
AGTCCGTTGAAGTGTCCGGCAGGATCCTCCAGATAAGAGCTTAGATAAGG
TCTGGGAAGGGTCCTGTCACATAATCTCCCACAATTCATCAACAACCACA
ATTCATCAATCCAATTAATTAACATCATCATCTCTCACAACAACAACAACA
ACATCAAATCATTCATAAACTCATTAATAAACAAAAAAAAAAAAAAAAAT
CTGGTTCTGGGTTCGCGAACCCAGAACCAGTGTCGATTCTGGGTTCACGA
ACCCAGAACCGATGTGGTTCTGGGTTCGCGAACCCAGAACCAGATCTGGT
TTTGGGCTCGGCGACGAGCCCAGAACCATATGGTTCTGGTTCGACCAGAT
CCAACTCAGGAAAAAAAAAAGAGAAAAAAAAGGAAAGAAGAAGAAGAAGA
AAAAGAAGAAGAAGAAGAGAGGGAGGAGGGATGAGAATATAGATGGGT
TAAGGCATTTTAGAGAGAGAATGAGAGGAAAAAATTTGATGGGGTGGGA
ATTTTAGAGAGAGAATCAGAAATGGGGTGGGTCATTGGAGGGAAAAACC
ATGGTAAGTGGTAATTAGCTGGACTATTAAGGGTATTTTGGATAAAAAAA
AATCATGCTGCAGGGAATTAAAAAAAAAAAGGTAATATCCCAAAAGGTTAT
AAAAAAAAGGTTAGATTTGGTTAAAACCCTTAAACAGAAAACTACAGATG
ATTGGTCTCTTTCAGAACTAAGAGAGGAAAACCAAGTCTTACACAAGAAA
GCTAGAGTCTTATCTATGTATTCTTGGTATCTGTTGAGTGGAAAATGTTTTT
GTTGCTTGTCTTGTCTATGTAGCAAGAACTGTGAAAATGTACTCTTTAGCC
TATGATATGTTTCCCAAACAACAAGAGTTGCAGCATTGTTTATGTAAGTAC
TTGGAAAAATTTTGTCTATTTATTATGCTTGTTATGAATAACAGTTTATGA
GATGCAACCATCACATATTCACATTGCTGTATGCTAGAGATGTGTGAAGC
CATTTAATTGGCCAAAATGGAGGCAACTTTTATTGGCTAAAAATTGAGGTT
AGCTTAAGCATGAAGAATTGTTATATATAAAAATCCCTTTTCCTCATCAAG
TAGCGGTCATTCCATTGTCCCAAGTCCTAATTCCTAGTGTAATTCTCTTCTC
TTTCCTTTAATTCTCTGCTAAAATTTTAGAAAGAGCTTGTAATTCTCTTGGT
TTAATAAAATATTTTTAGTTTTGGTTCACGAATGTTTCCTTTATATTAAAAA
TTTTATCATCTAAATTTTGTATTTACTATTTTTATATTATATTTTTTTTAATA
TTATTTTTAATTTTAAATTTACGTTGAGATTAGAATAATATCGATTTTTATC
ATTTTTATCATAGTGTAGTGTTTGGGAATATCATCATCAATCATGAGAAGG
CGCTGGAAAAGAGTACGCAAAATATGGCTGCAGTGATGCCTGCAGACAG
GCAAATAGTTTCTACCATGATTTACAGTAAATTGATTAGATTTTGATATGA
TCGACATTCTCAATTATCGATGAAGTAAATA**GCCTATTCCATGATCTTAA**
CGTGTGGTTATGTCAGCCTGTTACATATAGGTTTTTTTCCCCTTCATTA
TATGAGAGTTGATACCTATTAGCAGAGTCCAATG

<*LcF3H*-promoter

GTAAAGCAGTTGGCGACTACAAGTGCCAAGCAATCTCCCGCAATGGGAAT
AACATGACACATCATTTGGCTGTTTTGGTTCTTTCCTCTAGAGAAGATTTG
TCTGGGAGACAGTAAGCTTTGAATGTATTTTCCCTTTTTCTGGTTGTATAA
ATTTTTGTGTAATTTAAAAATATTTATATATCTATGTCATATTCAGTAAGC
ATTAATTACGCGATTGGGTACTTATCGGTACCGTCGTTCAAAACAATTCGA
TATTGAATAACTTTTATTCTGTTAAATAGTATTAGGTTCGAATAGTAATAT
TGTTAAATGGATATTAGAAAAATTATATGAATTAATGATGGAAGAATTAT
GCAACTACAAAGATTAGAGAAATTCTACAGTGGCATCTTTTTCAATTCTGA
TATAATTGCTTGAATCATTAATTTTAAAATTTAATAAATACTATTTAACAA
AAGTTATATCTAGTTGGTTAGCATCCCACAAACGTTAAACTTAAATGGTAT
GTATTAATGTTAATGATACTACATTAATTTAAGGGTTTTACTAATTTATTGT
TTGGGTAAGAATACCTATCAATAACAATCGTTGCATTAGATGCAAAGATT
GTTATTGGTGAATATTTAAGAATTATCATTAATGGATGTTTATAATTTTATT
TAACTAATAAGATAATAAACCTTTTAATTTAAATACACCACTTTAAAATTT
GATAATCTATTATTTTAATCAACTATTAACAAGAAAAAGCTTATTTATTTG
ATTATTTCCAATACCTGTATCTTTTCAGCTTTTAAATTAGGAACAAAAAAT
ATAAAATTGATGTTGAATTCAGAATTTAGTTATCTTTAAGAATTATAAATT
TAATTCCAAGCCACAATCCATTTATAGTGAATCTGAGCCTCCGATGTTTTT
TTTTTTAAATATTTAAATTAATTCATGAAAGAAATAAACATAATTAAACCT
ACAAACTCAATAATGCACCTATAAAACCATAGTACTAAATCAACTTCATT
AATTATGTTAACGCATTATAACATCAAGTCTTAAATACTATAGTTGGACTA
TTTTTTATCAGTTTAAACTTTATCAAAACTTAGAGGTCTTTAATTTAAACTC
TCTATCCATTTTATTCATCATCTTGTTGCACATGTTATACGGTAAGGAACA
TTTAGCGTGTGTCCATCATCTATTAACATGTTATACGGTAAGAAATATTTA
ACATATGTCTATCATTTGTTAACTG**TCCACGTTACACGTAAGACAACTTG**
TTGACATATTAGACAACGTGTTAACGTACCATAATCTCAAGTTTTAAATA
CTATAATTGGCTATTTTTCTATCAATTTAAGTTTTTAAAAATGATGATAACT
AACTAATTCAAACATATTTGTTTATAATTTGAAACAAAACAACATATTATG
AAACCAATGCAACCCTACTCGTCTTATATACAGGTGTGATTATGATTGAAC
CAGGTGGAATACGACATTTAAGTGAATATATAATCTCACTTTCCAAATAA
AATGAAGAGTGAATTCTAAAAAAACCTCCAACTTTTGCATATATATAAAA
AATAATAAAATAAAATAAAAACTCCAATATTTCGAAAATAAAGTTTTGCA
TCCTACCATTACACTTTCTATCTACTTCACCATTCACCATCCTTCTGAAAGT
TTTTATTTTCAAACAGAAATTCATAGAAAATTCTAACAGTGGGGTGCAAA
CCTTATATTTTCAAAAGGTCGGGTGTTTTTTTTATTTTCAGGCAAAAGGTG
GGGATGTTTTTAGAATTCACCCTAAAATGAATGAAAGAAAGTTATTCTAG
AAGCAATTAAGGGATTTATTTTGGTAGCTGTGGCATGAGCCATTGGAGGT
AGTTAGAGCTGCCACTCTCTAACCTTCCCTATCACTTACCTCTTTATATTTT
ATATAACTTCTCTCCTATAAAGCTACACTTACGCATCACACATTCGTTCAC
TCAAAACTTGCCTTCTCCACCACTAACTCAACCCTACAAACCAAGGCAAA
AAAAAAATCATACACTGATTAACACACCAAAAAAACAACTTGTGTGAATG

<*LcF3'H*-Promoter

AGAAAATGACAAGTGAAAAGGGCAGCAAAGTGGCTCCACCAGTTTATAT
AGGTGGCCGAGCTCGAGCCCCAGCCATCCGATTTGGAGAGTATGTAATTG
CCTTGAATCCAACGGTATAGCACGAGTAACGGCTTTGTAATCATGCAGCC
GTCAGTTGAAAGACGTGCACATTAAATGCGTGTGGCACGAGGCGTGCCTG
TGCTGGTTCAGTTATCTCCTGCATGATCGTCACATGTCAAAACTGATGGGA
TAGAATTTGAAAGAACGCGAAAACGACAATTCAAGACTAACTTTTACCAG
CGAGCTCAAGCTCCCAATTCGCAGAACGAACTGAGAGCTGGGGGGCTGAT
GTTATGCGTATTTTTTAGACTCAGCAAATGGGCTGGTAAAAGCCTGATTGA
AGCCCAACAAATCAACCCAATGAAGTGACTCAGCAGAAAAGAGCCGGTTT
ATCAATGCTAAGCCCAGCGAACTATACAGCTCGCACTAGCGAGCTCGCAT
CACATTGTTCTCAGATCGTTATGTAGTTCGCTCTATATACAACTTGCTAAT
CAGCGAGGCATAGCTCGAGATCGCTCAATACAATGTACAGCTCACTTAAC
GATCAGTTAGACAAGAAAATTTTTCAGAAAAGATAGTTACCCGATTTGAT
CGTGATTCTCTCATAAGAATTAAAAGTTAATTTAAATTTGAACTCAGTCAA
CATGATCTCGAATTTTACGGAGCAGTTTCCTGATTTGACTCAAACTGAATA
ACAAGTGCATTCAATGTGAAATTATTTTCTTGTTTTATTGCTACCTAAACT
GTAACGCCTCTTTCCCTATAAATAGAGGCTGAATTCATTATAAACAGATAT
ACAAACAATTCTATACTATCACTCTGTAGAATTTTTTAGAG**ACAGTCGTG**
GATGTAGGCGTACTCGCTGAATCACGTTAAAAACCTTGTGTCTTTTTTG
TGCTATTTTTATTCTTTGCCTTACTACTTGTTTGCGTGTTTTCTATTCTATTT
GAGATTGATTGTAGGTATTGGGATTTGAGCTCTCGTCGATTAGAAATCAAC
GTTAACTAGTATCAATTAATTAAACAACCAATATCAACTAATTTAAAGAA
AATATAAAAAGAAATGAAATCTAAATTAAAAATGAAATAACATCGAATTT
TGAAAAGAAATAATATTAAATTCTAAAATGAAATAACATCAAGTTCTAAA
ACGAAAGAATATGAAATCCTAAAATGAAATAATTCTAAATTAATAAAAAG
AAATAAAATAGGAAATGATCAAAAATATATCCCAATTAATTTAGAAAGTT
AAATCCTAATGAAATTTATTAAATAAAAATCTTAAATTAAACCAATTTTTT
AAATTAAACTGTGGTTGACTCTTTTTGATCCTCACAGGTTTACGAAAATTC
TTATAACAATATTTGAAAAAGTTAAAATCCAAATAAAAAGAAACAAAGCT
TGTATACATTAATTAATCACAACTTTGTCGTTTAAAACAGGAATTAGGATC
TGTAGATTTTTTGTTAATCTATGTAGTTATTGTAATGTGTAATTAATAGTTA
AGAATTTTTTTCTAAAAATATATTATGAACTTTATAGGTTTGGTTATTTTAT
TAATTAAGTAAGATTATTAATCAATAATAATTTATAAATTTTTTTTAATAA
TTATATTTATAATTAATTTAAGAGTTATTATTGATAAATATTTTATTCATTC
GAAGTGTAGATAGGCATAAATATAGGGAGAAGATTTATACACAGTGGATT
AAGTATTGAGGTCAAGGTACGATCAATTCTCCACCCCACCGGTCACCTGG
GCATAATTGAACTACCAAGGCATAATTGAACATTGAAAGCACGTGGTACC
TACCTCCTACGTGGCGTTTACCAAACAGCCTAACTCAAAACCCCATGTTTT
CACTTACCACTTCTTCACCTGTGCCATCCTCCACTCAGCCAAGCATGAACT
CCCGTATTTCCACTTGCCACTTGCTCACCTACGCACCTCTCTTCTCTTTTCT

ATATATGCACTGTCCTCCACTCGACTCCTCTCAACTCTACTAGGTAGTTTC
CCTCCTCCATCAATCCTTGTAACTAAAACTACTTTTCAATCAAAACATG

<*LcDFR*-promoter

AGTTGCCCAACATTTAGGGCAGATTTGCTTTGCACAATCGCATTTTCTCCT
TCTCACAGTCACAACATATAGAAATAAAATGTTAGCCTATATCAAGTATA
AATTAAAAGATTGAGTTTTGCCTCTTTAAATTGTATTGTTGCTGGTATTTTT
TTTTTTCCTTCGAGATATGCGTAGTTGAAAACACCTACATGTTGTGATTCG
CTACTATGATGACAGTCCATACAAAAAGAAATTAATATAAAAAAAGTTAT
AGTTTGGAGAGATACTAAACAATATGATGCAAAATTTCAACACAATAAGC
AATTCAAAATCCTTGAAATCCAACACGAAGGGAAATAAAAAAAAATGAA
AGAAGAAGAAAAAATTGCAAGTTTAACTGATACAAGACTTACATCTTCAT
TATTGTTTTTTTGCTTTTTCAACAAAAAGTCAAAAGAAGTTGGATCAAAAG
GGGGTTGACTCTTATCTCAACTAGGTTGACATCTGGTGAGTCCTTAAATCA
TGTGCGTGTAATCCTTCAACCGATCAACTTAAAACAAAAATAAAATACAA
AATAAGAGAACATAAACAAATTACAAACCTAAAAAAGTTGACAGAATCG
AAAAAAGGACAATGAAGACATGTATCTACCAACAAAAGAGCGATAAAAT
TCCGGACAGTTGAACCACTAACTATTAGTATTTAAGCGAAGACCATGAGA
AGCCAGAATATTTACAACTTGATTACTCTCATAATAAATATGAGTAACATG
ATAGTGCGTTTGAAAATATAGAACAAACATTTACAAGAGGCCAAAAGGTG
CCACGGAACATTGTGAAACTAAGTCTTTATTAAAGAAACCATATACAAGG
AATCACATTCAATCATAATAGAAGACCAAGAAAGCCATAAGCATACTCAA
TAGCTTTAATAACCGCTAGAATTTTAGCTTCATAAGCATATAAAATATATC
CCTATAACTAGAACAAATGGAGACCCATTTGAGGATCCATCAATGTTGAC
TTTAACCCAACCTGGAGGGGGGAGGATGCCAAAGTTGCAATGAACTTCCA
AAACACGTGCGGCACGATATATATGGACGAGGAGGAACATGCAAATTTCT
CAAATTAACAAACTTTATTGTATTTTTTTGTTGCAAATTATTTTCATGACTT
TGGAATAAAACAAAACTTTAAAATATTGATTAAAAAAAAGAGAGATATTT
TAGGTTTTGTAGAAAATAAAAGTGGAAGATAAACAATGTTGAATTCCAAA
ATAGGTCTATAGTGTCCTTTCTTTTTTCTCTCTATTAATTTTAAATTAGTTT
AAAATTATCATTTAAAAAAAAAAAAAAAAAAAAGGAGTGTGAAAATATAA
CTAGCATCACTTGTTTTGAACTTGTAGCACTAACAGTTTTAGTAATACAAT
TTATACAAAACCGTTGTGAAACGCCCTTGGTGCCCGATAAAGCCTTTCCTC
ACTAATAGAATTATATTACATATACACCAGTTTGAAAAGAAAATTATGGG
CACCGTGGGGTCCTCCGGCAGCTTCTATGTTCCCAATCACATCATATTATA
TTTGTGCTCCTATTCACTGAGCACAGGTGGTCCAAACTACAACCATTTAAA
GCTACAACATCTGTGGTTGTTAATGTCTCCATCCAAGCGAGAAGATTAGA
CTGAGCATGTGCCACCTA**CCACACTCGTGCCAAAATTGTTAGTTAACAA**
CGTGCCGGCCAGTCGGATGAGCTAAACCCCTACTGTGTTAGCTGGAAACT
CGGAATAAAAAGTTCCCTTTACGTCTACTATATATAAACAGCCCGACTCCG
ACAAAGTACCCCGAACATAGACGTCTCGCTTCACTTGTATCGATATATTTA

TAATCAGAATCATCAAAACTTGAAAAAGAAAAAGAAAAAAAGAAAAACA
TCATG

<*LcMYB1*-promoter

TCAAAGTCGAATGTCTTAGAAAGGAGACCCATTTTTCTGCTTAATTATTCT
ATTAACTCCATTACAATTATTTCAAGCCCAAAAGCAATTCCTAAGACATCA
AAAGTAAACATGCTACTATCATTAACAAAAGAAGAAAGAGATAAAAAGT
ACTACCAAAAAGTAGAGCCTCCTCCCAAATTTGGGATTATGCTAAAACAT
TATATTATATATTGACTACTTATTTATTTGTAAAATTTCAAATTAATCAAAT
GTCAGATACATATATATACATATATATATAATTATAGTGTAACCACTCAAA
CTTAGATCATTTTTTAATTAAATGAAAGTTCTATACCATCAAATCACATTA
AACAATATGGAATACATAATTACTTCTTACATTTTTATTCTCATTTCTAATT
CAAGAAGTAAAAGAATGTTCTATACATAGCTACAATCCAACTTGATTAAT
ATTTTTTGGGTTTTTGTTTATAATATATGTCCTTGGAGACTTCAGGATAGAT
AAAAATCACAAAAAATGAAAAACAACGCCAAGAAGATTTTTCACTGTCCT
AGAATTCATTGTTATGTGCTTAAATAAATCCCATATACTTTTCCCAAAAAA
AAAAAAATCCCATACAAAGATGTGTGGGATTGGTTGTATAGGATCGACTT
CAAAAAAGTTTTATTAGATAACAATATAATTCTCTTATTATTATTATTATT
ATTACTACTATTATTATTATTATTTTGTAATTATAGACATAATGGTAATTAT
ATTTATTTTCTTTAATCAATTTCAGAAAAGAAGGGAAAAAAGAGAATCTA
AAAGGCATTCACATCTTTCAATTTATACAAAACGATCCCATTGTTACATAT
CAGACAGATGTTAGTTATTATCATATTCAAAAGTTTAAACTGATAGAAAA
TTATCCAACTATAATATTTAAGACTTGAAGTTGACACGTGACATTCATTAT
ATCCAGCAAAATAATGAAAGTTAGCCAGTACACAAAGCATTAGGTTTTCT
GCCTAAACCTAATGCTCTCAATTACACAAAATAATGAAAATACTGTTGGC
TAGCAAGTTGCAAGTTTTGTAATTCGTTCTGTGTAAAGAAAGTTAGCTAGC
ACACAAAGCATTCCTCCTCCTATATACATAGAACTCTGTTTTGCAGAGTAA
CTATAGTAATTTACCGTACTATTTAGTTATTTGGTTTTCTGCCATAAGATCT
ACAACCCTACAAAATATCATGTGGTTAAGAGGAAAATTTTTAGCTGTACTT
CCAAGTGTGCGTGATAGTCATCAATTATTTCTCTTTAATTAAGAGATGCCT
ATTGATTATATTCTATGAGGAATTTAATTCTTGAATAAAAAATAGCCTTGA
AAGGTTAAGATACAATCAATAAACTTGATGAATATTCTCAATATTATTAGC
GTTAGAAAATGCCTTTGACATACTATCCAAAAGTTGTATTCAACCTTTTTT
TAAGAAAAACCTTATAAAGTAACTTTTTATTGTTTTCCAAAACTCAAACCA
TTTTGTATCTTCTGTTTCCAGAATAAACATGTTTAGAGAAAAGTCTCACCA
ATCATGTCATGTTGATAAACGGCTCTCATTTTCATTCTTTTTATTTATTATA
TTGTCGTTCATATAGACACATGTCGATCTGGGAATTGTTGGACTGGTAGTT
GACTTATAATGGATAGGGTCTGTTGTTAATCTTATACTCATGAAAGAACGC
GCAAACACTTCTTTCTAGTTTGTGGAGGCCAAGAAGAGCACGTCGTTCTAT
TTAAAGCTGTTGACGTATCCATTACTGAAGTCTTAGCGTGTGTCTATATAT
ACATACATATATATATATATAT**ATATATTTCGTGAGAGTACCTTATAAG**

CTCACACGATTATAGATATTAAAGCTCTATTGAGGTAGGCATGTCGCATT
TACTTGGGGCAAGTGCGTATAGTGCCACATCTATG

Figure S1. Nucleotide sequence of the promoters used in this work. The nucleotide sequence used in EMSA assay is indicated in bold. NAC core motif were indicated in red color. Translation start site (ATG) is shown in yellow box.

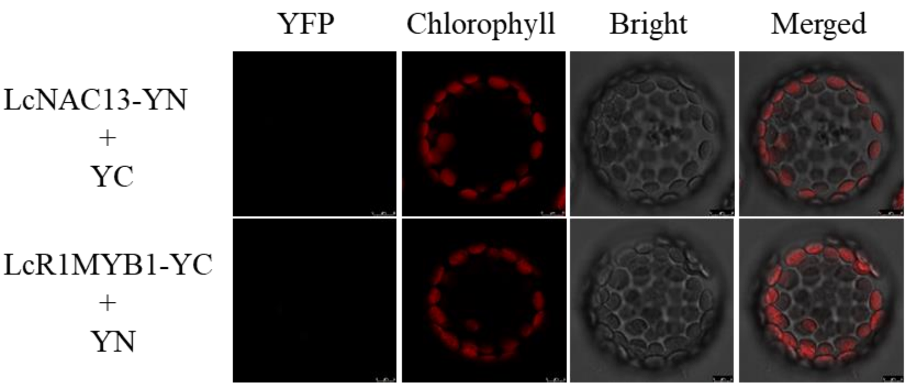

Figure S2. Bimolecular fluorescence complementation analysis. Fluorescence signal of YFP indicates protein-protein interaction. Combination of YN or YC with the corresponding LcNAC13 and LcR1MYB1 constructs were used as negative controls.

```
GmMYB176   ..........MSRASSAASGEIMLFGVRVVVD.................SMRKSVSMN     31
HvMCB1     .....ME..EEGGARK...AVLFRLFGVEVRG......AEEEEEDDAEP.MELKKSTSMP   43
HvMYBS3    MTRRCSHCSYNGHNSRTCPNRGVKIFGVRLTDG.................SIRKSASMG    42
LcR1MYB1   ..........................................................MN  2
OsMYBS2    .....MEQHEEAAERKPSPPVIFRLFGVEVRGGGGVDEEEYEEEEVEGGLFIKKSSSMP    55
OsMYBS3    MTRRCSHCSHNGHNSRTCPNRGVKIFGVRLTDG.................SIRKSASMG    42
ZmMYBSt1   MTRRCSHCSHNGHNSRTCPNRGVKIFGVHLTDGS................AIRKSASMG    43

GmMYB176   NLSQYE....HPLDA..TTTTNNKD..AVAAGYASADDAAPQNSGRHRERERKRGVPWTE   83
HvMCB1     NLASIG.....PILPRGEASASHDKGYASDDGELASTPQLKRRR..RKAQERKKGIPWTE   96
HvMYBS3    NLSLLG...GSTSGGGGASPADVGHDAA..AEGYASDDFVQGSS..SANRERKKGVPWTE   95
LcR1MYB1   NLSQYE....QPQEN..INSNSNKDDAAAAAGYASADDAVHNS...RASRERKRGVPWTE   53
OsMYBS2    NLTSID.....PLP....VPADGGKRRASDDSELASG.QQKRRR..RKVQERKKGVPWTE   103
OsMYBS3    NLSLLSSAAGSTSGG..ASPADGPDAAPTAADGYASDDFVQGSS..SATRDRKKGVPWTE   98
ZmMYBSt1   NLSLLS..AGSTSGG..ASPADGPDLAD.GGGGYASDDFVQGSS..SASRDRKK.VFLGL   95

GmMYB176   EEHKLFLVGLQKVGKGDWRGISKNYVKTRTPTQVASHAQKYFLRRSNLNRRRRRSSLFDI   143
HvMCB1     EEHRKFLEGLKQLGKGDWRGISKNFVTTRTATQVASHAQKYFLRQTNPGKKKRRASLFDV   156
HvMYBS3    EEHRRFLLGLQKLGKGDWRGISRNFVVSRTPTQVASHAQKYFIRQANMSRRKRRSSLFDL   155
LcR1MYB1   EEHKLFLLGLQKVGKGDWRGISRNFVKTRTPTQVASHAQKYFLRRSNLNRRRRRSSLFDI   113
OsMYBS2    EEHKKFLEGLRQLGKGDWRGISKNFVTSRTATQVASHAQKYFLRQTNPGKKKRRASLFDV   163
OsMYBS3    EEHRRFLLGLQKLGKGDWRGISRNFVVSRTPTQVASHAQKYFIRQSNMTRRKRRSSLFDM   158
ZmMYBSt1   EKNTGVFAGITKARERGWRGISRNFVVSRTPTQVASHAQKYFIRQSNMSRRKRRSSLFDM   155

GmMYB176   TTDTVSAIPMEEEQVQNQDTLCHSQQQPV.........FPAETS.KINGFPAMPVYQFGV   193
HvMCB1     G......IPAGHGYDDQLPSPHSVGTKLA...........PAEKILHTDRGDVPLPSYS.   198
HvMYBS3    VPDES.DLPPLPGNHEPEAQILNHPPLPPPMEEEEVESMESDTSVIAESSSASAIMPENL   214
LcR1MYB1   TTDSVTAIPMEEEQESNK.TRSHPLPPPP.........LPETNKGGVSGFSVIPAFPV..   161
OsMYBS2    .........VAECSDDQLPSPQSVGTKP............PTQDIIHTDRGDVPILSY..   200
OsMYBS3    VPDESMDLPPLPGGQEPETQVLNQPALPPPREEEEVDSMESDTSAVAESSSASAIMPDNL   218
ZmMYBSt1   VPDESMDLPPLPGSQEPETSMLNQPPLPP.AVEEEVESMESDTSAVAESSGASALMPESL   214

GmMYB176   GSSGVISVQGTGNPMEELTLGQGNVEKH...NVPNKASTVSGIITPGSSSSAIDP.....   245
HvMCB1     .....EVIRGNNNNNTQMQADELTDHAKK.........RSKFPTGMSLTAMAASGL....   240
HvMYBS3    QPSYPVLVPAYFSPFLQFSVPFWQNQNDGDDLGQGTHEIVKPVPVHSKSPINVDELVGMS   274
LcR1MYB1   ....VLPVP.VENPIDSLTLGQPNQSND...APANLIRPIPVLPAPQPSAVVSDLNL...   210
OsMYBS2    .....PVARGFRGD..SVQVDELTEYVK.........RLKAAEDMSLSMISG.......   236
OsMYBS3    QSTYPVIVPAYFSPFLQFSVPFWQNQKDEDGPVQETHEIVKPVPVHSKSPINVDELVGMS   278
ZmMYBSt1   QPTYPMIVPAYFSPFLQFSVPFWPNQEDGGDLPQETHEIVKPVAVHSQNPINVDELVGMS   274

GmMYB176   ......PPTLSLGLSFSSDQRQTSSRHSALHAMQCFS.NGDS..IISV              284
HvMCB1     ......ELAMSSSPSSILELSIGPPRHQAVD.AIKVL..........               270
HvMYBS3    KLSIGDPKQDTVSTSLSLKMVGGQNRQSAFQANLPTRAQA........              314
LcR1MYB1   NLNMVDPSSLSLKLSLPSDQREASSRHSAFQAMPSLSSNGDSGSIISV              258
OsMYBS2    .......LEMASSSISSLELSIAPP.HQAIEAAIKVL..........               265
OsMYBS3    KLSIGESNQETVSTSLSLNLVGGQNRQSAFHANPPTRAQA........              318
ZmMYBSt1   KLSIWEHGQETVYTSLSLNLLGGQNRQSAFHANPQTRAQA........              314
```

Figure S3 Sequence alignment of LcR1MYB1 with other plant R1-MYB proteins. The highly conserved SHAQK(Y/F) F motifs is underlined.
